# A high-spatial-resolution dataset of human thermal stress indices over South and East Asia

**DOI:** 10.1038/s41597-021-01010-w

**Published:** 2021-09-01

**Authors:** Yechao Yan, Yangyang Xu, Shuping Yue

**Affiliations:** 1 School of Geographical Sciences, Nanjing University of Information Science & Technology, Nanjing, Jiangsu China; 2 Department of Atmospheric Sciences, College of Geosciences, Texas A&M University, College Station, Texas USA

**Keywords:** Climate-change impacts, Atmospheric science

## Abstract

Thermal stress poses a major public health threat in a warming world, especially to disadvantaged communities. At the population group level, human thermal stress is heavily affected by landscape heterogeneities such as terrain, surface water, and vegetation. High-spatial-resolution thermal-stress indices, containing more detailed spatial information, are greatly needed to characterize the spatial pattern of thermal stress to enable a better understanding of its impacts on public health, tourism, and study and work performance. Here, we present a 0.1° × 0.1° gridded dataset of multiple thermal stress indices derived from the newly available ECMWF ERA5-Land and ERA5 reanalysis products over South and East Asia from 1981 to 2019. This high-spatial-resolution database of human thermal stress indices over South and East Asia (HiTiSEA), which contains the daily mean, maximum, and minimum values of UTCI, MRT, and eight other widely adopted indices, is suitable for both indoor and outdoor applications and allows researchers and practitioners to investigate the spatial and temporal evolution of human thermal stress and its impacts on densely populated regions over South and East Asia at a finer scale.

## Abstract (summary)

**Table:** 

Measurement(s)	thermal stress
Technology Type(s)	computational modeling technique
Factor Type(s)	temporal interval • geographic location
Sample Characteristic - Environment	climate system
Sample Characteristic - Location	South Asia • East Asia

Machine-accessible metadata file describing the reported data: 10.6084/m9.figshare.15149010

## Background & Summary

Due to the unprecedented scale of climate change, extreme temperature events have become more intense and frequent in many parts of the world over the past few decades^1–3^. The study of thermal stress or discomfort due to heat or cold extremes has attracted attention worldwide^4–7^, as thermal stress can have a pronounced negative impact on human health, especially in vulnerable populations such as the elderly, chronically ill and poorer communities^8–10^.

The level of human thermal stress is determined not only by the ambient air temperature (*T*_*a*_) but also by a combination of other factors, including solar and thermal radiation (*R*), wind speed (*V*_*a*_), relative humidity (*RH*), personal clothing and activity level. To date, more than 100 indices have been developed to assess and quantify human thermal stress^11,12^. These indices vary considerably in type, complexity, and capability. Some of them are based on the principles of human thermal exchange, while others are based on empirical relationships obtained by examining human responses to various environmental factors.

Many of the empirical indices, such as the heat index (HI), humidity index (Humidex), net effective temperature (NET) and wind chill temperature (WCT), use only two or three environmental parameters (e.g., *T*_*a*_ and *RH*) and thus are only applicable to indoor space or outdoor shaded areas. However, some classic indices, due to their simple form and low data input for computation, remain attractive and are widely utilized by national and local weather services^13^.

A few human thermal stress indices, such as the universal thermal climate index (UTCI), the standard effective temperature (SET), and the physiological equivalent temperature (PET), consider more meteorological factors, allowing them to be used in both indoor and outdoor conditions. The UTCI, which is the focus of our study, is a state-of-the-art thermal stress indicator based on heat budget models of the human body and its surrounding environments^14^. The UTCI takes into account a suite of relevant meteorological variables (air temperature and humidity, wind speed, and longwave and shortwave radiant heat fluxes) as well as personal factors such as physical activity level and adaptive clothing behaviour, making it applicable in a variety of climates, seasons and spatial scales^15–17^.

Using the key variables from ERA5-Land reanalysis, along with direct solar radiation from ERA5, this paper presents a higher-spatial-resolution (0.1° × 0.1°) gridded dataset with multiple thermal-stress indices. This newly developed dataset, called the High-spatial-resolution Thermal-stress Indices over South and East Asia (HiTiSEA), contains daily maximum, minimum, and mean values of the indoor and outdoor UTCI (including shaded and unshaded outdoor environments), as well as the mean radiant temperature (MRT) and eight other widely used empirical indices, as listed in Table 1, from 1981 to 2019 for the area of South and East Asia, a region making up more than half of the world’s population, many of which are vulnerable to the impacts of extreme thermal stress. Another reason for the limited spatial coverage is due to data access issues, as observed meteorological data used in this study for validation are only available in this area.

**Table 1:** Thermal indices and their input variables.

Thermal Indices	Full Name of the Indices	Air Temperature	Air Humidity	Wind Speed	Radiation
UTCI	universal thermal climate index	*T*_*a*_	*e*	*V*_*a*_	*R*
indoor UTCI	UTCI for indoor environment	*T*_*a*_	*e*		
outdoor shaded UTCI	UTCI for outdoor shaded space	*T*_*a*_	*e*	*V*_*a*_	
MRT	mean radiant temperature				*R*
ESI	environment stress index	*T*_*a*_	*RH*		*SR*
HI	heat index	*T*_*a*_	*RH*		
Humidex	humidity index	*T*_*a*_	*e*		
WBGT	wet-bulb globe temperature	*T*_*a*_	*e*		
WBT	wet bulb temperature	*T*_*a*_	*RH*		
WCT	wind chill temperature	*T*_*a*_		*V*_*a*_	
AT	apparent temperature	*T*_*a*_	*e*	*V*_*a*_	
NET	net effective temperature	*T*_*a*_	*RH*	*V*_*a*_	

Note: *T*_*a*_, *e*, and *RH* represent the air temperature, water vapour pressure, and relative humidity, respectively. *V*_*a*_ stands for the 10-metre wind speed, with the exception of the NET (Eq. 12), which requires an input of wind speed at 1.2 m above the ground. *R* stands for the radiation variables, including direct, diffuse, and reflected solar radiation, as well as upward and downward thermal radiation, while *SR* represents the solar radiation, which includes both the direct and diffuse solar radiation reaching the horizontal surface of the Earth. The indoor UTCI, outdoor shaded UTCI, and UTCI, which take 2, 3, and 4 parameters, respectively, are applicable to indoor, outdoor shaded, and outdoor unshaded environments. All indices are with a unit expressed in °C.

Compared to the existing 0.25° × 0.25° spatial-resolution ERA5-HEAT (Human thErmAl comforT) product^18^ released by the European Centre for Medium-Range Weather Forecasts (ECMWF) and the Human Discomfort Indices (HDIs, also with a spatial resolution of 0.25° × 0.25°) computed from the Global Land Data Assimilation System (GLDAS) by Mistry^19^, the new features of the HiTiSEA dataset include the following:

(i) It features a higher spatial resolution (0.1° × 0.1°, but smaller spatial coverage) based on ERA5-Land reanalysis;
(ii) It contains 3 types of UTCI indices (UTCI, indoor UTCI, and outdoor shaded UTCI), MRT metrics, and eight other empirical thermal indices that allow applications for indoor, outdoor shaded, and outdoor unshaded environments;
(iii) It provides comprehensive validation based on thousands of weather stations over South and East Asia (including bias and root mean square error for each index at each station released as part of the dataset), which enables users to further evaluate and select some indices over the others and conduct bias correction if needed;
(iv) It shares freely available Python scripts that allow users to calculate the UTCI and its variants, as well as other thermal indices for any part of the world.

With a finer spatial resolution and a wider applicability to both indoor and outdoor conditions, this multiple-index dataset is a valuable resource for health authorities and scientists to study the evolution of the thermal environment and identify high-risk areas where people are exposed to potential heat or cold stress. Tourism professionals will find it useful in evaluating thermal comfort conditions and defining the most appropriate time for specific recreational activities. These data can also be used by researchers and policy makers to assess the costs of extreme thermal stress on the economy through reduced labour productivity. Moreover, this newly developed dataset can help researchers estimate the energy demand required to meet residential heating or cooling needs, especially in India, Bangladesh, and China, where large gaps exist^20^.

## Methods

### Data source

A complete set of meteorological data, including air temperature and humidity, wind speed, and shortwave and longwave radiation fluxes, is required for computation of the thermal indices included in the HiTiSEA dataset. While various reanalysis products, such as the Global Land Data Assimilation System Version 2 (GLDAS-2) developed jointly by the National Aeronautics and Space Administration (NASA) and National Oceanic and Atmospheric Administration (NOAA), the Modern-Era Retrospective analysis for Research and Applications, Version 2 (MERRA-2) produced by NASA, the Japanese 55-year Reanalysis (JRA-55) released by the Japan Meteorological Agency (JMA), etc., are currently available, the ERA5 and ERA5-Land datasets developed by the ECMWF are chosen for use in the present study, as other reanalysis products have either (i) coarser spatial resolutions (e.g., the GLDAS-2, MERRA-2, and JRA-55 provide gridded meteorological variables with a horizontal resolution of 0.25° × 0.25°, 0.625° × 0.5°, and 1.25° × 1.25° in longitude/latitude, respectively) or (ii) incomplete meteorological variables (e.g., direct solar radiation is not available in other reanalysis products).

ERA5 is the fifth-generation atmospheric reanalysis product recently released by the ECMWF. ERA5 is generated using the latest version of the Integrated Forecasting System and modern parameterizations technique, with a horizontal resolution of 0.25° × 0.25°, a temporal resolution of 1 h, and a vertical resolution of 137 levels from the surface up to a height of 80 km^21,22^. By rerunning the land component of the ERA5 climate reanalysis, the ECMWF has developed a state-of-the-art reanalysis dataset called ERA5-Land, which covers the land surface of the entire globe with a horizontal resolution of 0.1° × 0.1° and a temporal resolution of 1 h. Using “lapse rate correction”, ERA5 air temperature, air humidity and pressure used to run ERA5-Land are corrected to account for the altitude difference between the grid of the forcing and the higher-resolution grid of ERA5-Land^23^.

Due to storage limitations, HiTiSEA version 1 presented in this study spanned the period from 1981 to 2019, covering the area of East Asia and South Asia (65°E to 155°E and 3°N to 58°N). To compute the MRT and UTCI, hourly meteorological variables (Table 2) were retrieved from ERA5-Land, with the exception of *fdir* (direct solar radiation at the surface), which is only available in ERA5. Since the variable *fdir* has a coarser resolution, it is regridded from 0.25° × 0.25° to 0.1° × 0.1° using nearest-neighbour interpolation to match the other variables. The nearest-neighbour method is used due to its advantage in preserving the values of the original data^24^. Other resampling methods, such as bilinear and cubic convolution, can increase uncertainties by altering or even distorting the grid values of the original data. Furthermore, the accumulated radiation values in the original dataset of ERA5-Land (J m^−2^, as in Table 2) are transformed to hourly values. Note that the convention for accumulations used in ERA5-Land differs from that for ERA5^25^.

**Table 2:** Variables from ERA5-Land and ERA5 to compute MRT and UTCI.

Variable	Description	Units	Source Dataset
*T*_*a*_	The temperature of the air at 2 m above the ground	K	ERA5-Land
*T*_*d*_	Dewpoint temperature at 2 m above the ground	K	ERA5-Land
*u*	Eastward component of the 10 m wind	m s^−1^	ERA5-Land
*v*	Northward component of the 10 m wind	m s^−1^	ERA5-Land
*ssrd*	Surface solar radiation downwards: the amount of shortwave radiation (both direct and diffused) that reaches a horizontal plane at the surface	J m^−2^	ERA5-Land
*ssr*	Surface net solar radiation, the amount of shortwave radiation (both direct and diffuse) that reaches a horizontal plane at the surface minus the amount reflected at the surface	J m^−2^	ERA5-Land
*strd*	Surface thermal radiation downwards: the amount of thermal (longwave) radiation emitted by the atmosphere and clouds that reaches a horizontal plane at the surface	J m^−2^	ERA5-Land
*str*	Surface net thermal radiation: the difference between downward and upward thermal radiation passing through a horizontal plane at the surface	J m^−2^	ERA5-Land
*fdir*	Direct solar radiation at the surface: the amount of direct shortwave radiation passing through a horizontal plane at the Earth’s surface, which is equal to the *ssrd* but excluding the diffused solar radiation.	J m^−2^	ERA5

### Data processing procedure

**Fig. 1:**
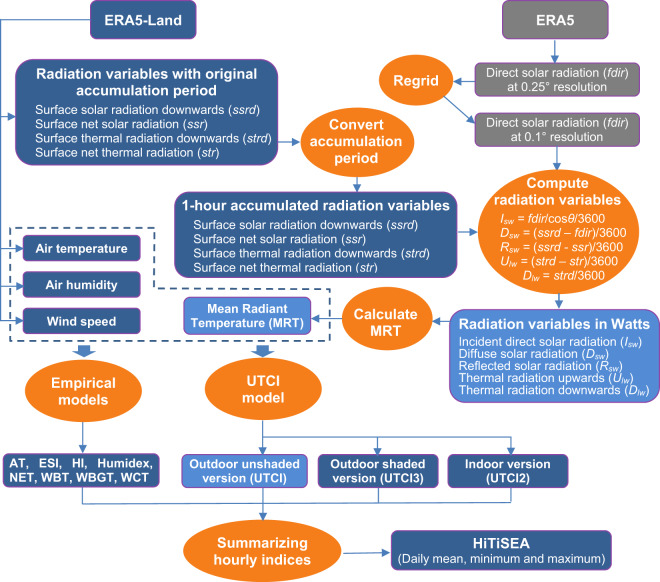
Schematic of the workflow to generate the HiTiSEA product.

Figure 1 shows the procedure for processing the ERA5-Land and ERA5 reanalysis data and producing the multi-thermal-index dataset. The procedure includes the following five steps: (1) extracting the variable of direct solar radiation from ERA5 and regridding it from 0.25° to 0.1°; (2) extracting other radiation variables from ERA5-Land and converting the accumulated radiations to hourly accumulated values; (3) computing the radiation variables, expressed in W/m^2^, in the MRT formula (Eq. 1); (4) calculating the MRT; (5) computing the indoor and outdoor UTCI as well as other empirical thermal indices on an hourly basis; and (6) performing summary statistics for these hourly indices and archiving the HiTiSEA dataset with daily mean, maximum and minimum values.

### Calculation of MRT

The MRT is defined as the effective temperature of an imaginary enclosure in which the radiant heat transfer from the human body equals the actual radiant heat transfer in the real nonuniform enclosure^26^. MRT is the key parameter used to compute UTCI. It is used to assess the impact of radiation fluxes on the energy balance of human bodies, which is not accounted for in indices such as Tw. In operational human biometeorology, fluxes are related to an upright standing or walking person^27^. Since a resolution of 0.1° × 0.1° or approximately 10 km is insufficient to capture the details of individual persons’ surrounding environment, an unshaded plain is assumed with solid angles (*f*_*a*_) of the land surface and the sky both set to 0.5. Then, the MRT for the outdoor environment is given by Weihs *et al*.^28^.

1 $$MRT={\left\{\frac{1}{\sigma }\left[\frac{{\alpha }_{k}}{{\varepsilon }_{p}}\left({f}_{p}\cdot {I}_{sw}+{f}_{a}\cdot {D}_{sw}+{f}_{a}\cdot {R}_{sw}\right)+{f}_{a}\cdot \left({D}_{lw}+{U}_{lw}\right)\right]\right\}}^{0.25}-273.5$$

where *MRT* is the mean radiant temperature (°C), *σ i*s the Stefan Boltzmann constant (5.67 × 10^−8^ W m^−2^ K^−4^), *α*_*k*_ is the absorption coefficient of the typical human body for shortwave radiation (here assuming standard value 0.7), and *ε*_*p*_ is the emissivity coefficient of the human body (here assuming standard value 0.97). *I*_*sw*_, *D*_*sw*_, *R*_*sw*_, *D*_*lw*,_ and *U*_*lw*_, all expressed in W/m^2^ and calculated following the equations in Fig. 1, are the anisotropic incident (*I*_*sw*_) direct shortwave radiation flux, isotropic diffuse (*D*_*sw*_) shortwave radiation flux, surface reflected (*R*_*sw*_) shortwave radiation flux, downwelling (*D*_*lw*_) longwave radiation fluxes and upwelling (*U*_*lw*_) longwave radiation fluxes, respectively.

The projected area factor (*f*_*p*_) accounts for the directional dependence and is a function of the solar zenith angle. For a rotationally symmetric standing human body, *f*_*p*_ can be estimated using the following formula^29,30^:

2 $${f}_{p}=0.308\cdot \cos \left\{\left(\frac{\pi }{2}-\theta \right)\cdot \left[1-\frac{{\left(90-\frac{180}{\pi }\theta \right)}^{2}}{48402}\right]\right\}$$

where *θ* is the solar zenith angle (in radians). The cosine of the solar zenith angle can be calculated following Woan^31^:

3 $$\cos \,\theta =\sin \,\delta \,\sin \,\varphi +\cos \,\delta \,\cos \,\varphi \,\cos \,h$$

where *φ* is the geographical latitude, *δ* is the solar declination angle as a function of a given date of the year and *h* is the hour angle in local solar time. The latter two parameters, i.e., *δ* and *h*, are calculated following Spencer^32^ and NOAA^33^.

Considering that *I*_*sw*_ can be overestimated at sunset and sunrise times (note that it is computed by dividing *fdir* by cos*θ*, which is close to 0 during those twilight periods), the average cos*θ* between the beginning of the forecast time and the end of the forecast step (1-hour interval in this case) is used instead of the exact endpoint of the forecast time. A detailed description for calculating the average cos*θ* can be found in Di Napoli *et al*.^34^.

### Calculation of UTCI

The UTCI is defined as an equivalent ambient temperature (in the unit of °C) of a reference environment that produces the same physiological response of a typical person as in the actual environment^14^. Calculation of physiological response to meteorological inputs is based on an advanced multinode thermoregulation model (consisting of 12 body elements with a total of 187 tissue nodes) coupled with an adaptive clothing model considering behavioural changes in clothing insulation related to the actual thermal environment^15,16^. The reference environment^14^ is defined as a condition with calm air (a 10-m wind speed of 0.5 m/s), where the mean radiant temperature equals the air temperature, a 50% relative humidity is used for Ta ≤ 29 °C, and a water vapour pressure *e* = 20 hPa is used for Ta > 29 °C, where an average person walks at 4 km/h, generating a metabolic rate of 135 W/m^2^.

Due to our need to produce a climate dataset with high spatial and temporal resolutions, calculating the UTCI by repeatedly running the thermoregulation model is not practical. In this study, a 6th-order polynomial regression function given by Bröde *et al*.^35^ is used to calculate the outdoor, unshaded UTCI. The simple form of the function is written as follows (with the full equation in the code release):

4 $$UTCI={T}_{a}+f({T}_{a},\;{V}_{a},e,MRT-{T}_{a})$$

where *T*_*a*_ is the 2-metre air temperature, *V*_*a*_ is the 10-metre wind speed (m/s), *e* is the water vapour pressure (hPa), and *MRT* is the mean radiant temperature (°C).

To compute the outdoor shaded UTCI, *MRT* is set equal to the air temperature, thus ignoring the radiation flux’s contribution to thermal comfort. To compute indoor UTCI, in addition to *MRT, V*_*a*_ is also set to the reference values of 0.5 m/s, thus further ignoring the ambient wind speed’s contribution to thermal comfort.

### Calculation of other empirical thermal indices

#### Apparent Temperature

The apparent temperature (AT) is defined as the temperature at the reference humidity level, producing the same amount of discomfort as that experienced under the current ambient temperature, humidity, and solar radiation^36^. Two forms are in use by the Australian Bureau of Meteorology: one includes radiation and one does not. The AT index used here is based on a mathematical model of an adult walking outdoors in the shade^37^ and thus does not include radiation:

5 $$AT={T}_{a}+0.33\times e-0.7{V}_{a}-4$$

where *AT* is the apparent temperature (°C), *T*_*a*_ is the air temperature (°C), *e* is the water vapour pressure (hPa) and *V*_*a*_ is the 10-m wind speed (m/s).

#### Environment Stress Index

The environmental stress index (ESI) was introduced by Moran *et al*.^38^ in 2001 as a substitute for the wet bulb globe temperature (WBGT), which was hard to use due to the required measurements of nonconventional meteorological variables such as the wet-bulb temperature and global temperature. The ESI, which was validated by using large databases and was found to be highly correlated with the WBGT^39^, is calculated as^38^:

6 $$ESI=0.63T-0.03RH+0.002SR+0.0054\times T\times RH-\frac{0.073}{0.1+SR}$$

where *T* is the air temperature (°C), *RH* is the relative humidity (%), and *SR* is the amount of solar radiation (both direct and diffused, in W/m^2^) that reaches a horizontal plane of the Earth’s surface.

#### Heat Index

The heat index (HI) is widely used across the United States. It is a measure of how hot it feels when relative humidity is factored in along with the air temperature. The original heat index is a hot-weather version of AT that involves a collection of equations and a large number of input parameters^40^. To arrive at an equation that uses more conventional independent variables, a regression equation was obtained by Rothfusz^41^ through multiple regression analysis based on the data from Steadman’s table:

7 $$\begin{array}{lll}HI & = & -42.379+2.04901523\times {T}_{a}+10.14333127\times RH\\  &  & -0.22475541\times {T}_{a}\times RH-0.00683783\times {T}_{a}^{2}-0.05481717\times R{H}^{2}\\  &  & +0.00122874\times {T}_{a}^{2}\times RH+0.00085282\times {T}_{a}\times R{H}^{2}\\  &  & -0.00000199\times {T}_{a}^{2}\times R{H}^{2}\end{array}$$

where *HI* is the heat index (in °F), *T*_*a*_ is the temperature (in °F) and *RH* is the relative humidity (in %).

If the *RH* is less than 13% and the temperature is between 80 and 112 °F, then the following adjustment is subtracted from *HI*:

8 $$Adj=\frac{13-RH}{4}\times \sqrt{\frac{17-\left|{T}_{a}-95\right|}{17}}$$

On the other hand, if the *RH* is greater than 85% and the temperature is between 80 and 87 °F, then the following adjustment is added to *HI*:

9 $$Adj=\frac{RH-85}{10}\times \frac{87-{T}_{a}}{5}$$

The Rothfusz regression is not suitable when the *HI* is below 80 °F. In those cases, a simpler formula, provided by the National Oceanic and Atmospheric Administration^42^, is applied to produce values consistent with Steadman’s results:

10 $$HI=0.5\times \left[{T}_{a}+61+\left({T}_{a}-68\right)\times 1.2+RH\times 0.094\right]$$

#### Humidex

The Humidex (short for humidity index) is an index developed by Canadian meteorologists^43^ to describe how hot the weather feels to the average person. By combining the effects of air temperature (*T*_*a*_, in °C) and water vapour pressure (*e*, in hPa), the Humidex (in °C) is calculated as follows:

11 $$Humidex={T}_{a}+0.5555\times (e-10)$$

#### Net Effective Temperature

The net effective temperature (NET) was originally established in 1923 by Houghton and Yaglou^44^ to estimate the relative effects of air temperature and humidity on body comfort. It was amended, based on laboratory experiments, by Missenard^45^ using the empirical relationship between the identical state of the organism’s thermoregulatory capacity (warm and cold perception) and differing temperature and humidity of the surrounding environment. However, Missenard’s formula seemed exclusively appropriate for hot weather conditions. Further modifications included the effect of winds and extended its use to cold conditions^46,47^. The resulting formula takes the following form:

12 $$NET=37-\frac{37-{T}_{a}}{0.68-0.0014\times RH+\frac{1}{1.76+1.4\times {V}_{a}^{0.75}}}-0.29\times {T}_{a}\times \left(1-0.01\times RH\right)$$

where NET is the net effective temperature (°C), *T*_*a*_ is the air temperature (°C), *RH* is the relative humidity (%) and *V*_*a*_ is the wind speed (m/s) at a height of 1.2 m, which is approximated by applying a typical logarithmic wind profile approach:

13 $${V}_{a}={V}_{{Z}_{r}}\frac{\log \left(Z/{Z}_{0}\right)}{\log \left({Z}_{r}/{Z}_{0}\right)}$$

where *Z* is the height (m) of the centre of the body element above ground (i.e., 1.2 m in this case), *V*_*Zr*_ (m) is the wind speed at a reference height of the meteorological measurement (i.e., 10 m), and *z*_0_ (m) is the roughness length, assumed to be 0.01 m^16^.

#### Wet-Bulb Globe Temperature

The wet-bulb globe temperature (WBGT), developed in the 1950s by the US Navy as part of a study on heat-related injuries during military training, is one of the most widely used heat stress indices throughout the world. The WBGT is a composite temperature in which the natural wet-bulb temperature *T*_*w*_ (°C), the black globe temperature *T*_*g*_ (°C), and the dry-bulb temperature *T*_*d*_ (°C) are added up with different weightings according to their importance^48^:

14 $$WBGT=0.7\times {T}_{w}+0.2\times {T}_{g}+0.1\times {T}_{d}$$

Due to the lack of *T*_*w*_ and *T*_*g*_ in ERA5-Land, the WBGT is calculated using a simplified equation, given by the Australian Bureau of Meteorology^49^, as follows:

15 $$WBGT=0.567\times {T}_{a}+0.393\times e+3.94$$

where *T*_*a*_ is the air temperature (°C) and *e* is the water vapour pressure (hPa).

This simplified equation, which only takes the air temperature and the water vapour pressure into consideration, is only applicable for indoor environments.

#### Wet Bulb Temperature

The wet bulb temperature (WBT) is the lowest temperature that can be reached under current ambient conditions through the evaporation of water. At 100% relative humidity, the WBT is equal to the air temperature, while at a lower humidity, it is lower than the air temperature due to the effect of evaporative cooling. In practice, WBT is measured using a wet-bulb thermometer. In this paper, WBT is approximated using Stull’s formula^50^:

16 $$\begin{array}{lll}WBT & = & {T}_{a}\times {\rm{atan}}\left[0.151977{\left(RH+8.313659\right)}^{0.5}\right]+{\rm{atan}}\left({T}_{a}+RH\right)-{\rm{atan}}\left(RH-1.676331\right)\\  &  & +0.00391838{\left(RH\right)}^{1.5}{\rm{atan}}\left(0.023101\times RH\right)-4.686035\end{array}$$

where *WBT* is the wet bulb temperature (°C), *T*_*a*_ is the air temperature or dry bulb temperature (°C) and *RH* is the relative humidity (%). The approximation is valid for relative humidity ranging from 5% to 99% and air temperature from −20 °C to 50 °C.

#### Wind Chill Temperature

The wind chill index (WCI), developed in the 1940s and revised by weather services in the USA and Canada, expresses the enhancement of heat loss in cold climates from exposed body parts due to wind. In the present study, the WCT was calculated using a multiple regression formula developed by the Joint Action Group for Temperature Indices^51^. The following formula provides the equivalent temperature (what the temperature feels like to the human body when the cooling effect of wind is taken into account) as an output:

17 $$WCT=13.12+0.6215\times {T}_{a}-11.37\times {V}_{a}^{0.16}+0.3965\times {T}_{a}\times {V}_{a}^{0.16}$$

where *T*_*a*_ is the air temperature (in °C) and *V*_*a*_ is the 10-m wind speed (in km/h).

## Data Records

The geographically gridded dataset consists of daily mean, maximum and minimum values of the following thermal indices at a 0.1°× 0.1° spatial resolution: (1) the universal thermal climate index for the unshaded outdoor environment (UTCI); (2) the universal thermal climate index for shaded outdoor space (outdoor shaded UTCI); (3) the universal thermal climate index for the indoor environment (indoor UTCI); (4) the apparent temperature (AT); (5) the environmental stress index (ESI); (6) the heat index (HI); (7) the Humidex; (8) the mean radiant temperature (MRT); (9) the net effective temperature (NET); (10) the wet bulb temperature (WBT); (11) the wet bulb globe temperature (WBGT); and (12) the wind chill temperature (WCT).

The dataset spans the period from January 3, 1981, to December 31, 2019, covering the area of South and East Asia (65°–155°E, 3°–58°N). Individual thermal stress indices were aggregated into a single NetCDF file on a daily basis. Each daily file is named as follows:

$${\rm{H}}{\rm{i}}{\rm{T}}{\rm{i}}{\rm{S}}{\rm{E}}{\rm{A}}{\rm{\_}}{\rm{Y}}{\rm{Y}}{\rm{Y}}{\rm{Y}} \mbox{-} {\rm{M}}{\rm{M}} \mbox{-} {\rm{DD.}}{\rm{n}}{\rm{c}}$$

where “YYYY-MM-DD” represents the date of the daily file.

The variables are named in the following format: Index_mean, Index_max and Index_min. For example, the variables for the daily mean, maximum and minimum of UTCI are named UTCI_mean, UTCI_max and UTCI_min, respectively. For each variable, grid cells with no data are filled with the value −32767.

This newly developed dataset^52^, with a total volume of 450 GB, contains 14242 daily NetCDF files that are archived by year and compressed into tar.gz files to save storage space. The dataset and its metadata are freely available at the figshare repository (10.6084/m9.figshare.c.5196296).

## Technical Validation

We select nine indices in our dataset (Table 3) for comparison, which do not require radiation data for computation. They were compared against the corresponding indices computed from observed meteorological data obtained from the China Meteorological Data Service Center (CMDSC)^53^ through a portal located at Nanjing University of Information Science & Technology (NUIST)^54^. The observed data in 2018 have a temporal resolution of 3 h, including the air temperature (*T*_*a*_), dew-point temperature (*T*_*d*_), 10-metre wind speed (*V*_*a*_), and surface air pressure (*P*). Meteorological records with missing or incomplete values (missing any of the above 4 meteorological variables) were excluded, and 1281 stations were finally used for validation.

**Table 3:** Summary table of accuracy, in terms of RMSE (°C) and bias (°C), obtained by comparing the indices computed from ERA5-Land reanalysis and weather station observations.

Thermal Indices	Daily Mean		Daily Maximum		Daily Minimum	
	RMSE	Bias	RMSE	Bias	RMSE	Bias
indoor UTCI	1.6	−0.4	1.9	−0.7	2.2	−0.3
outdoor shaded UTCI	2.7	−0.9	3.1	−1.2	3.7	−0.7
HI	2.0	−0.6	2.4	−0.9	2.5	−0.4
Humidex	1.9	−0.6	2.3	−0.8	2.7	−0.5
WBGT	1.1	−0.4	1.3	−0.5	1.6	−0.3
WBT	1.3	−0.3	1.4	−0.4	1.9	−0.3
WCT	3.1	−1.7	4.8	−2.5	3.3	−1.3
AT	2.0	−0.7	2.3	−0.9	2.7	−0.7
NET	2.7	−0.3	3.3	−0.7	3.6	0.2

This table only lists the indices that do not require radiation as data input.

**Fig. 2:**
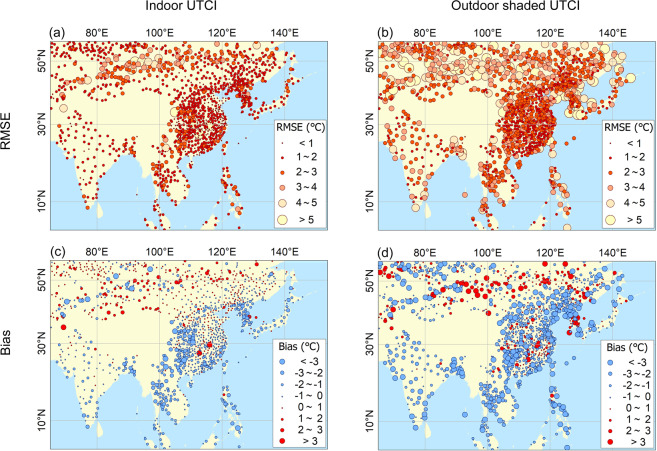
Spatial distribution of values of RMSE and bias for daily mean indoor UTCI (left column) and outdoor shaded UTCI (right column) computed from ERA5-Land.

Table 3 shows that the RMSE values for daily mean, maximum, and minimum indoor UTCI are 1.6 °C, 1.9 °C, and 2.2 °C, respectively, with 81% of the stations presenting an RMSE for daily mean lower than 2 °C (Fig. 2 upper left), making this index ideal for indoor thermal stress assessment. In comparison, the outdoor shaded UTCI shows higher RMSE values, with approximately 30% of the stations having an RMSE for daily mean less than 2 °C and 71% having an RMSE below 3 °C. Stations with RMSE values greater than 5 °C, as depicted in Fig. 2 (upper right), are mostly located in higher-latitude areas and a few coastal areas where the wind speed is significantly affected by local factors. As depicted in Fig. 2 (lower row), both the estimated indoor UTCI and outdoor shaded UTCI are overall negatively biased, with more stations exhibiting negative bias and fewer stations, most of which are located north of the line of latitude 40°N, exhibiting positive bias.

Among the empirical thermal indices with 2 climate parameters, the WBGT shows the highest accuracy, with RMSE values ranging from 1.1 to 1.6 °C, followed by the WBT ranging from 1.3 to 1.9 °C. HI and the Humidex, which also take air temperature and air humidity as input variables, present RMSE values no more than 2.5 °C and 2.7 °C, respectively. The WCT with input variables of air temperature and wind speed, however, shows the lowest accuracy, with RMSE values varying between 3.1 °C and 4.8 °C. For the 3-parameter empirical thermal indices, the average RMSE values for daily mean, maximum and minimum AT are found to be 2.0 °C, 2.3 °C, and 2.7 °C, respectively, and the RMSE values for NET are all above 2.7 °C but no more than 3.6 °C.

Almost all indices listed in Table 3 are slightly biased towards negative values, which suggests that compared to the observed results, these thermal-stress indices are underestimated in most cases. While on average, the bias for estimation of daily maximum WCT can be as large as −2.5 °C, the biases for most indices are within −1 °C.

The other three indices, i.e., the MRT, the outdoor unshaded UTCI, and the ESI, which require radiation for calculation, were also evaluated against the corresponding indices computed from observations but with a much smaller sample. This is because hourly radiation data are not open to the public and are difficult to acquire. While commonly observed meteorological variables (i.e., *T*_*a*_, *T*_*d*_, *V*_*a*_, *P*, etc.) are all available at the 1281 stations with a time step of 3 h, and only 8 of them provided daily radiation observations for 2018 to registered users on CMDSC’s website. The observed radiation data include daily values of global radiation, direct solar radiation, diffuse solar radiation, reflected solar radiation, maximum global radiation flux, the time when maximum global radiation flux occurs, etc. To assimilate the two sets of observations with different time steps, we rounded the time when the maximum global radiation flux occurred to the nearest 3-hour synoptic time (00:00, 03:00, 06:00 UTC, etc.). By doing so, we paired the maximum global radiation flux with the commonly observed meteorological data. After removing incomplete records, these paired-up observations have a size of 2220 hourly records, as listed in Table 4 for each station. They were then fed into the BioKlima 2.6 software package^55^ to calculate the MRT and the outdoor unshaded UTCI for those specific records. These observational results were used to validate the corresponding hourly MRT and UTCI from which the daily maximum, minimum, and mean MRT and UTCI were derived in our dataset. Similarly, the paired-up radiation data and other meteorological data were input into Eq. (6) to compute the observational ESI for validation of the corresponding ESI in our dataset.

**Table 4:** Average RMSE values (°C) and biases (°C) of the MRT, UTCI, and ESI for stations that have both radiation data and other commonly observed meteorological data for 2018.

Station ID	Station Name	Longitude	Latitude	Number of Records	MRT		UTCI		ESI	
					RMSE	Bias	RMSE	Bias	RMSE	Bias
54511	Beijing	116.47	39.80	230	10.1	8.1	5.4	3.8	1.0	−0.1
54342	Shenyang	123.52	41.73	283	8.7	4.3	4.5	0.1	1.6	−0.2
50953	Harbin	126.57	45.93	282	11.1	8.0	5.5	2.9	1.5	−0.3
58362	Baoshan	121.45	31.40	289	7.4	3.3	3.2	−0.5	1.2	−0.7
57494	Wuhan	114.05	30.60	284	9.8	5.4	3.8	0.7	1.6	−0.4
59287	Guangzhou	113.48	23.22	288	7.1	3.6	2.9	0.5	1.5	−1.0
56187	Wenjiang	103.87	30.75	289	9.9	2.2	3.9	0.9	1.9	−1.3
51463	Urumqi	87.65	43.78	275	12.1	1.6	6.9	−0.8	3.2	−0.4

Compared to the existing ERA5-HEAT product, which has an RMSE of 5.2 ± 2.5 °C^18^, this newly developed outdoor unshaded UTCI, due to the use of the enhanced resolution of ERA5-Land, exhibits improved accuracy with an average RMSE of 4.5 °C, ranging from 2.9 °C to 6.9 °C (Table 4). However, using finer resolution radiation data from ERA5-Land does not seem to have a significant effect on the accuracy of the MRT, which has an average RMSE of 9.5 °C with a range of 7.1 °C to 12.1 °C, compared to the MRT (with an RMSE of 8.6 ± 2.5 °C) released along with the UTCI in the ERA5-HEAT product. This is partly because the direct solar radiation, which is the most important radiation variable in determining the MRT, is derived from ERA5, not ERA5-Land. Another reason that leads to the low accuracy of the MRT might be due to the small number of radiation stations used for validation (Table 4). In contrast, the ESI shows strong consistency with the observational result (RMSE values at 7 out of 8 stations are all below 2 °C), which suggests that the outdoor thermal-stress indicator of the ESI is not as sensitive to the change in solar radiation as the UTCI.

Concerning the biases of the three indices listed in Table 4, while the MRT exhibits strong positive biases and the ESI shows slight negative biases at all stations, the UTCI, however, has inconsistent results, with 6 stations positively biased and 2 negatively biased.

**Fig. 3:**
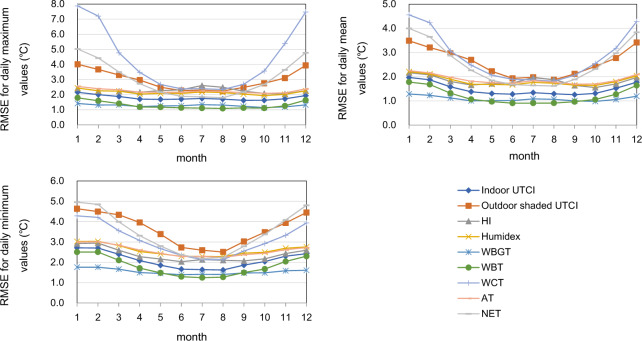
Average monthly RMSE values for daily maximum (upper left), minimum (lower left), and mean (upper right) thermal-stress indices. This figure only includes the nine indices that don’t require radiation as data input.

**Fig. 4:**
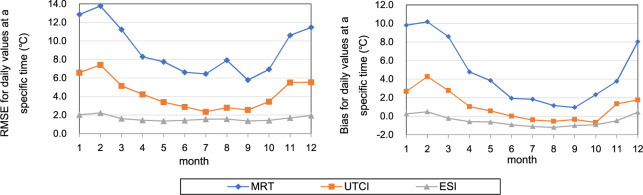
Average monthly RMSE values (left) and biases (right) for daily values of the MRT, UTCI, and ESI at specific time of the day when maximum global radiation flux occurs.

Because the accuracy of weather forecasts varies throughout the year, the reliability of this dataset differs in different seasons. Generally, the dataset has a better performance in warm periods and summer monsoon seasons than on cold winter days (Figs. 3 and 4). This is especially true for those indices that include the variables of wind speed or radiation. For example, the RMSE for daily mean values of the outdoor shaded UTCI ranges from the lowest value of 1.9 °C in August to the highest value of 3.5 °C in January. The accuracy of the WCT, which uses wind speed and air temperature for calculation, shows the strongest seasonal effect, with the RMSE for daily maximum values varying between 2.4 °C and 7.9 °C. The accuracy of AT and the other two-variable indices with air temperature and humidity as inputs (i.e., the indoor UTCI, HI, Humidex, WBGT, and WBT), however, exhibits a slight seasonal effect, with RMSE values for the daily mean, maximum and minimum ranging from 1.0 °C to 2.3 °C, 1.1 °C to 2.6 °C and 1.3 °C to 3.0 °C, respectively, in the validation year.

**Fig. 5:**
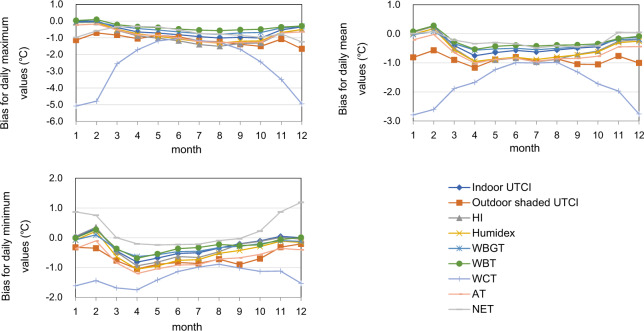
Average biases for daily maximum (upper left), minimum (lower left), and mean (upper right) thermal-stress indices. This figure only includes the nine indices that don’t require radiation as data input.

As seen from Figs. 4 and 5, while most of the indices are negatively biased across all seasons, the MRT is positively biased throughout the year, especially in cold winter months. The UTCI is biased towards positive values most of the year except for July to October.

To enable users to learn more about the seasonal effects of dataset accuracy at individual weather stations, we created text-formatted validation files (archived and named “validation.tar.gz”, available at the abovementioned repository) in which the monthly and yearly summaries of RMSE and bias at each station, as well as their locations, are included. With these data, users can reduce uncertainties by examining the verification results at stations located in their study areas.

## Usage Notes

**Fig. 6:**
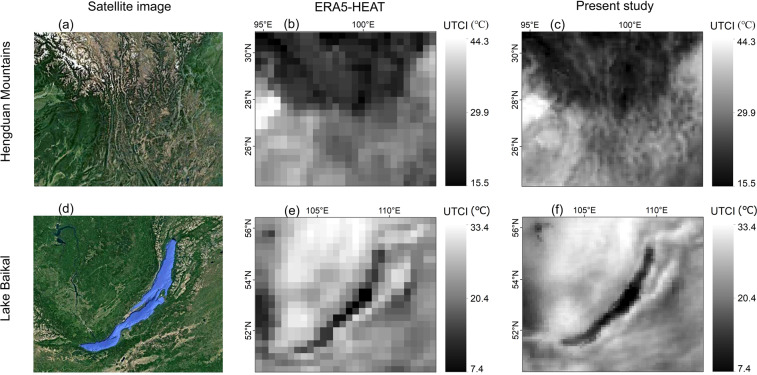
The satellite images from Google Earth for the regions of Hengduan Mountains (upper left) and Lake Baikal (lower left), and the distributions of daily maximum UTCI from ERA5-HEAT (middle) and the present study (right) on 2018-07-20.

In comparison to the existing 0.25° × 0.25° spatial resolution thermal-index product^18^, this dataset provides more details on studying the spatial variation of heat/cold stress. As seen from the upper images in Fig. 6, the 0.1° × 0.1° gridded UTCI allows us to quantify the difference between the human thermal stress in longitudinal valleys and their associated mountain ridges in Southwest China. The lower images of Fig. 6 show that while the spatial contrast of UTCI near Lake Baikal is blurred in the 0.25° × 0.25° gridded product (downloaded from the Copernicus Climate Data Store implemented by ECMWF), more detailed information, especially along the lakeshore, is visible in our new dataset.

Combined with heat- or cold-related morbidity and mortality, this dataset can be used to identify thermal stress thresholds for the general population or specific groups working indoors or outdoors. This dataset can also serve to assess the thermal comfort conditions required for tourism activities directly exposed under the sun or in the shade.

**Fig. 7:**
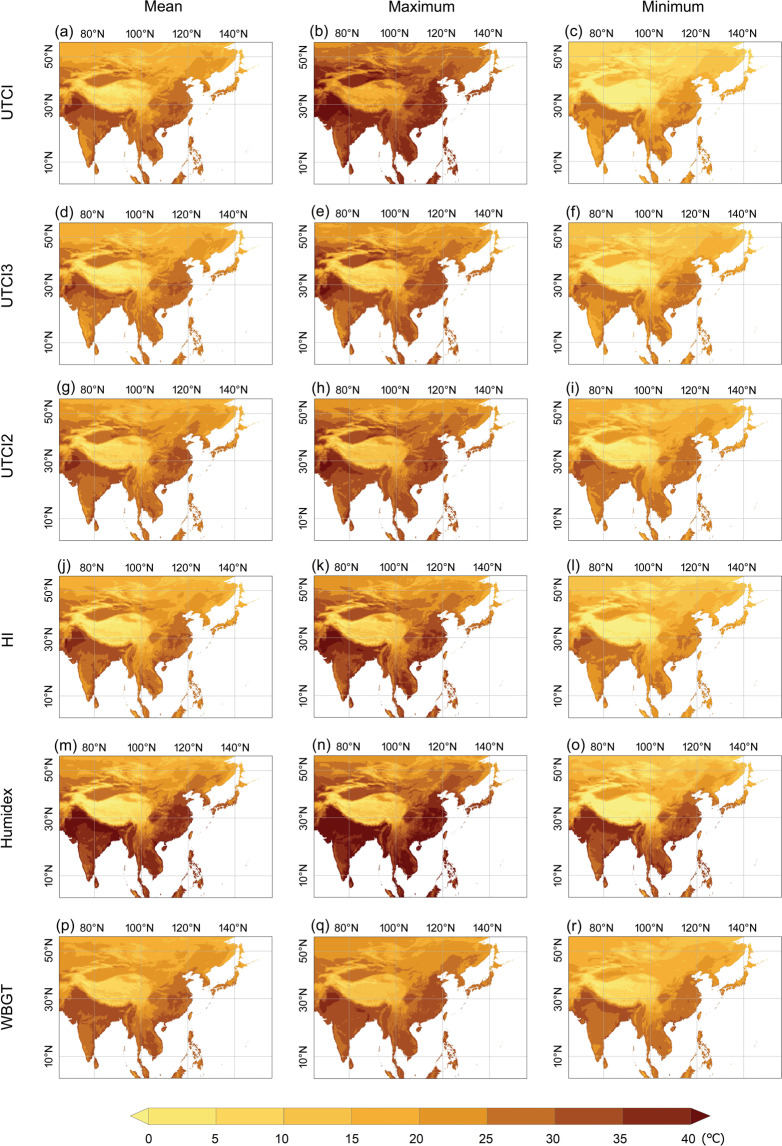
Averaged daily mean (left column), maximum (middle column), and minimum (right column) of the thermal indices for July during the period of 1981 - 2019. Only select indices suitable for hot conditions are illustrated. UTCI2 refers to the indoor UTCI, which uses two parameters, and UTCI3 stands for the outdoor shaded UTCI, which takes three parameters for the calculation.

**Fig. 8:**
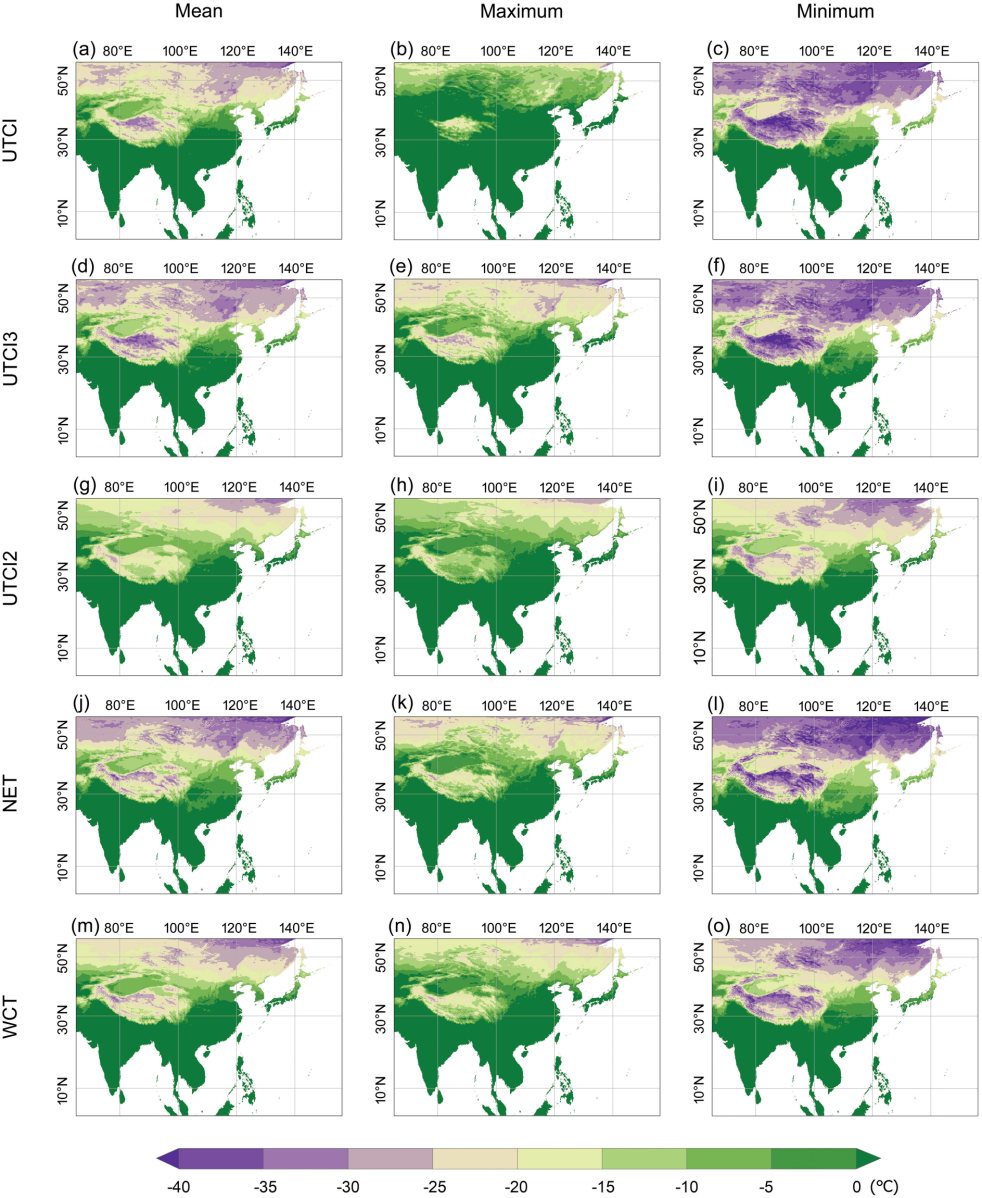
Averaged daily mean (left column), maximum (middle column), and minimum (right column) of the thermal indices for January during the period 1981 - 2019. Only essential indices suitable for cold conditions are illustrated. UTCI2 refers to the indoor UTCI, which uses two parameters, and UTCI3 stands for the outdoor shaded UTCI, which takes three parameters for the calculation.

Although all thermal indices used in this study are temperature equivalents expressed in degrees Celsius (note that a conversion from Fahrenheit to Celsius for the index of HI is performed) and share a similar spatial pattern (Figs. 7 and 8), it is worth noting that each index is associated with a particular assessment scale. For example, UTCI values between 32 °C and 38 °C are categorized as “strong heat stress”^35^, whereas for Humidex, a similar sensation would range from 40 °C to 45 °C^43^. A comprehensive description of assessment scales with defined thresholds for commonly used thermal indices was provided by Blazejczyk *et al*.^11^.

Another important note is that while the UTCI can be applied in all climates and all seasons throughout the year, the use of the other indices is often restricted to specific conditions. For example, two-variable indices (with air temperature and humidity as inputs), such as the indoor UTCI, HI, Humidex, WBGT and WBT, are suitable for use in indoor conditions, while three-variable indices, such as the outdoor shaded UTCI, AT and NET, can be applied in an outdoor shaded environment, as the effect of wind speed is accounted for.

While this dataset shows higher accuracy in flat areas (e.g., the Indo-Gangetic Plain and the lowland plains in eastern China, as shown in Fig. 2), its accuracy degrades in areas with heterogeneous landscapes, especially in mountainous areas (e.g., western mountainous areas of China), with strong orographic effects and coastal zones affected by the mixed-pixel problem (e.g., areas along the coastline of the Korean Peninsula where land and water coexist within specific grid cells). Researchers and practitioners interested in those regions might have to pay more attention, as thermal-stress indices may vary substantially due to complex topography or land-water contrasts at a subgrid scale.

## Data Availability

All codes for calculating the indoor and outdoor UTCI, MRT, and other empirical thermal indices, written in Python (3.8) using cdsapi (0.3.1), numpy (1.19.2), pandas (1.1.3), netCDF4 (1.5.4), and scipy (1.5.3) libraries, were developed on Linux (CentOS 6.10) and can be easily adapted to Windows and other platforms. The codes are freely available at the abovementioned repository^52^.
